# Corrigendum: Adrenergic-mediated loss of splenic marginal zone B cells contributes to infection susceptibility after stroke

**DOI:** 10.1038/ncomms16151

**Published:** 2017-08-18

**Authors:** Laura McCulloch, Craig J. Smith, Barry W. McColl

Nature Communications 8 Article number: 15051 ; DOI: 10.1038/ncomms15051 (2017); Published: 04 19 2017; Updated: 08 18 2017

The affiliation details for Barry W. McColl are incorrect in this Article. The correct affiliation details for this author are given below:

The Roslin Institute and R(D)SVS, University of Edinburgh, Easter Bush, Midlothian EH25 9RG, UK.

UK Dementia Research Institute, University of Edinburgh, Edinburgh Medical School, 47 Little France Crescent, Edinburgh EH16 4TJ, UK.

