# Supplementary material for: Adrenergic-mediated loss of splenic marginal zone B cells contributes to infection susceptibility after stroke
Source: Nat Commun. 2017 Apr 19;8:15051. doi: 10.1038/ncomms15051 (PMC5399306; doi:10.1038/ncomms15051)
Supplement: Supplementary Information — Supplementary Figures and Supplementary Table [file ncomms15051-s1.pdf]

a

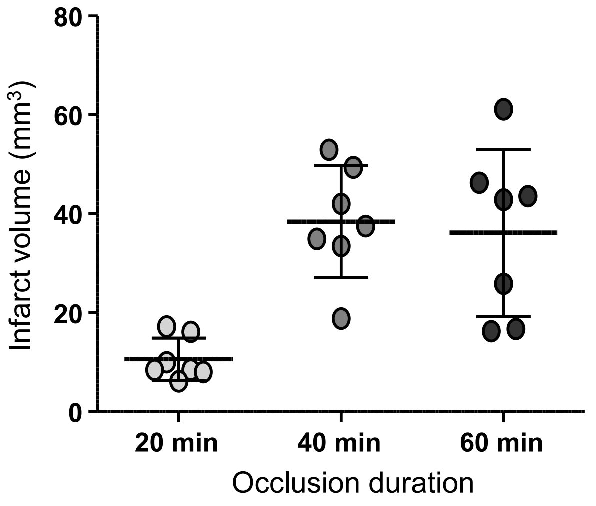


b

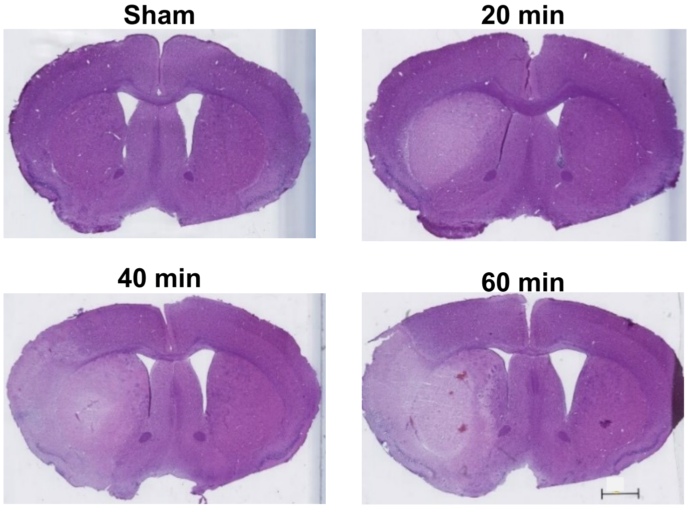


**Supplementary Figure 1**
(**a**) Quantification of infarct volume 48h after different durations of transient MCAO (n=7). Data show mean ± SD (**b**) Cresyl violet staining of brain sections illustrates the typical extent and distribution of brain damage induced by each duration of MCAO. 20 min MCAO causes injury largely restricted to the dorsolateral striatum whereas 40 min and 60 min MCAO cause moderate to extensive damage in both cortical and subcortical regions. No damage is evident in the brains of sham-operated mice. Scale bar, 1mm.

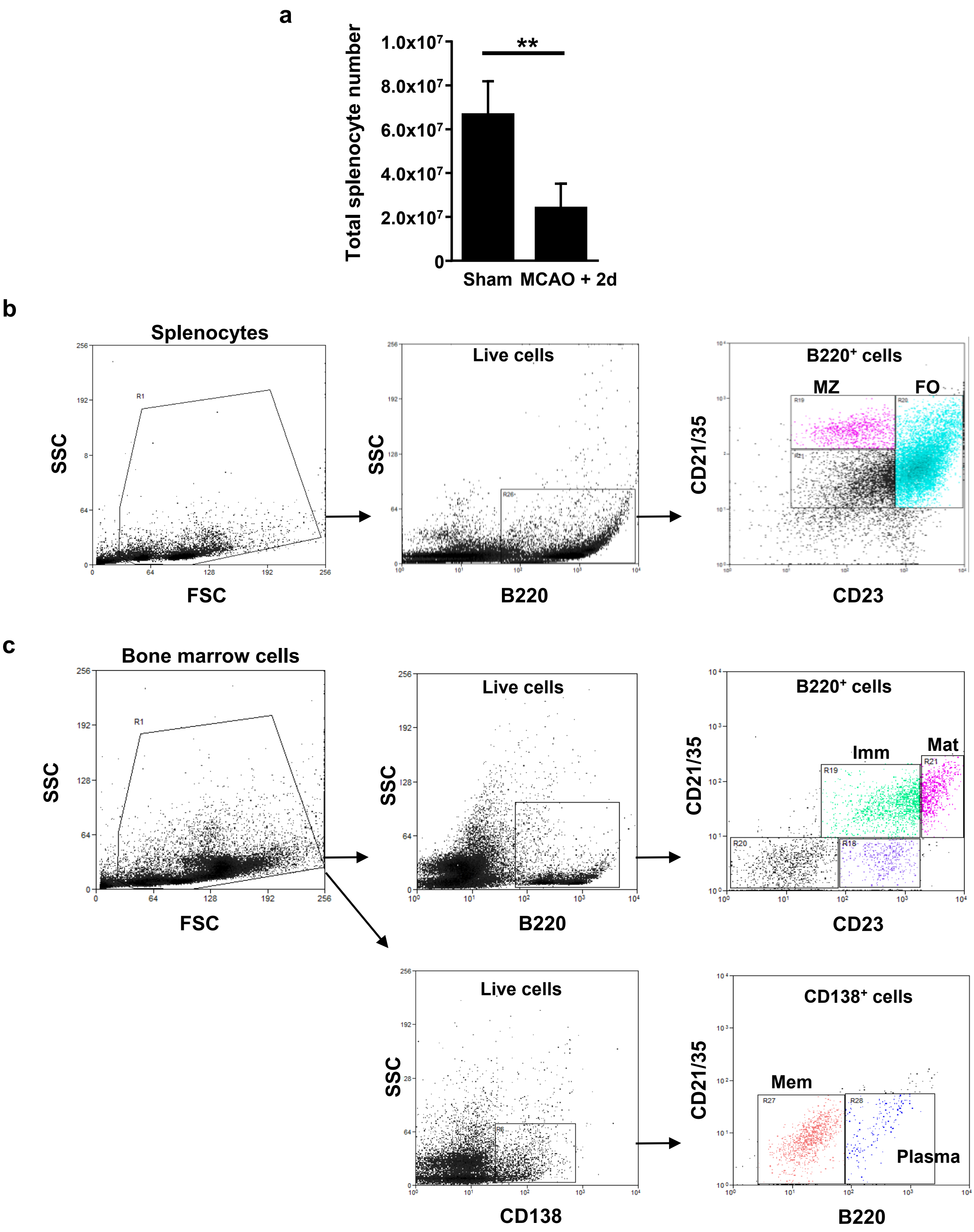


**Supplementary Figure 2**

(**a**) Total splenocyte counts show a 65% reduction 2 d after MCAO in comparison to sham surgery (sham n=4, MCAO n=6). Data show mean + SD; **P<0.01; unpaired t-test. (**b**) Gating strategy for detection of splenic marginal zone (MZ, $B220^{+}CD23^{-}CD21/35^{hi}$) and Follicular (FO, $B220^{+}CD23^{+}CD21/35^{int}$) B cells. (**c**) Gating strategy for detection of bone marrow B cell subsets. Immature B cells (Imm) were identified as $B220^{+}CD23^{-}CD21^{+}$ and mature recirculating naive B cells (Mat) as $B220^{+}CD23^{+}CD21^{+}$. Memory B cells (Mem) were identified as $CD138^{+}B220^{-}CD21^{-/lo}$ and plasma B cells were identified as $CD138^{+}B220^{-}CD21^{-/lo}$.

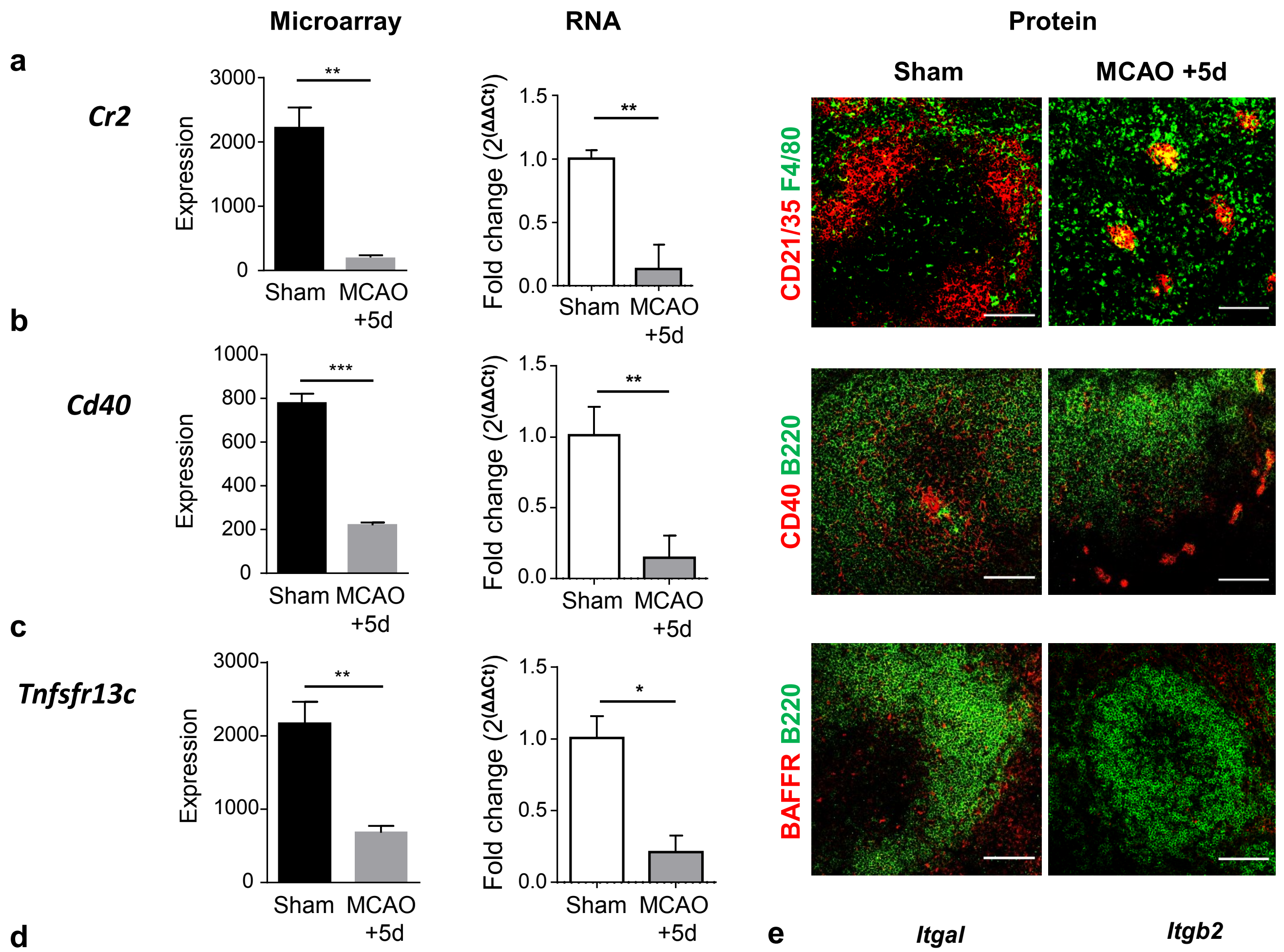


d

| Term | P Value |
|---|---|
| Hematopoietic cell lineage | $1.0 \times 10^{-10}$ |
| Primary immunodeficiency | $3.3 \times 10^{-8}$ |
| B cell receptor signalling pathway | $2.1 \times 10^{-7}$ |
| Toll-like receptor signalling pathway | $7.6 \times 10^{-6}$ |
| Natural killer cell mediated cell cytotoxicity | $1.8 \times 10^{-5}$ |
| Cell adhesion molecules | $3.2 \times 10^{-5}$ |
| T cell receptor signalling pathway | $1.1 \times 10^{-4}$ |
| Intestinal immune network for IgA production | $2.0 \times 10^{-4}$ |
| Cytokine-cytokine receptor interaction | $2.0 \times 10^{-4}$ |
| Chemokine signalling pathway | $2.0 \times 10^{-4}$ |

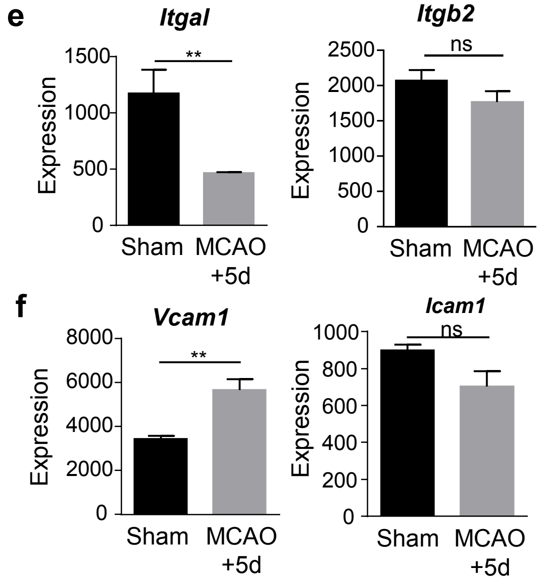


**Supplementary Figure 3**

Microarray data for selected B cell-related genes were validated at RNA level by qPCR. Reduced expression of *Cr2* (**a**), *Cd40* (**b**) and *Tnfsfr13c* (**c**) RNA was confirmed by qPCR. A loss of CD21/35 (encoded by *Cr2* gene) immunolabelling (**a**) in the MZ (arrows) and reduced FDC immunolabelling in the follicle and reduced co-localization of CD40 (red, **b**) and BAFFR (red, **c**) with B220$^+$ B cell immunolabelling (green, **b, c**) in mice 5 d after MCAO in comparison to sham surgery validated these changes at protein level (*Sham n=4, MCAO + 5d n=5*). (**d**) Transcripts identified as downregulated after MCAO were analysed in KEGG to identify affected pathways. Pathways associated with the development and function of various aspects of the immune system were identified as deficient after MCAO. (**e**) Microarray expression levels for transcripts of the integrin LFA-1 (*Itgal* and *Itgb2*), important for retention of B cells in the MZ, are decreased in spleens after MCAO in comparison to sham-operated controls. (**f**) In contrast, transcripts for the adhesion molecules these integrins bind to, expressed by stromal cells (*Vcam1* and *Icam1*) are increased/ unchanged after MCAO (*sham n=2, MCAO n=3*). Scale bars **a, b, c** 200 μm. Data show mean ± SD; **a, b, c, e, f** unpaired *t*-test; ns not significant, *$P$<0.05, **$P$<0.01, ***$P$<0.001.

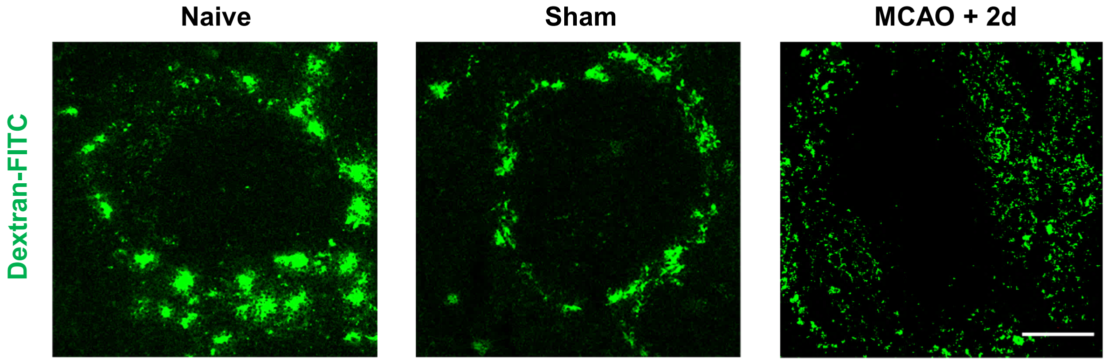


**Supplementary Figure 4**

Fluorescently labelled model antigen (green, Dextran-FITC) is trapped in the spleens of naïve animals and animals recovered 2 d after MCAO or sham-surgery, 1 h after i. v. injection.

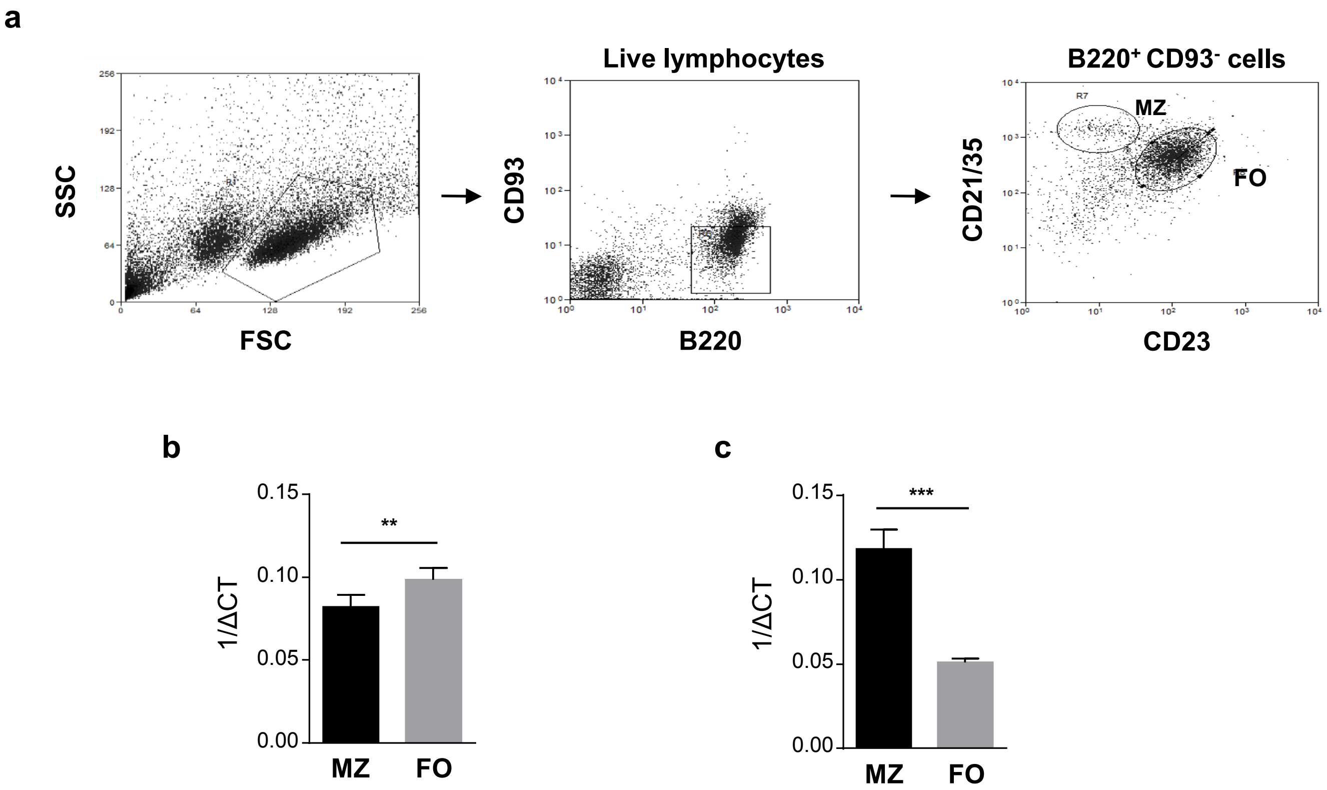


**Supplementary Figure 5**

The expression levels of *Adrb2* were determined by qPCR analysis of RNA extracted from marginal zone (MZ) and follicular (FO) B cell populations sorted by FACS from a mixed splenocyte suspension. (**a**) MZ (B220$^+$CD93$^-$CD23$^-$CD21/35$^{hi}$) and FO (B220$^+$CD93$^-$CD23$^+$CD21/35$^+$) B cells were obtained by cell sorting . (**b**) Expression levels of *Adrb2* were slightly higher in FO B cells in comparison to MZ B cells. (**c**) Expression of *Cd1d*, encoding a non-classical MHC protein expressed by MZ B cells, was expressed at a significantly higher level in RNA extracted from MZ compared to FO B cells confirming the enrichment of MZ B cell RNA. n=9. Data show mean ± SD; unpaired *t*-test, $^{**}P<0.01$, $^{***}P<0.001$

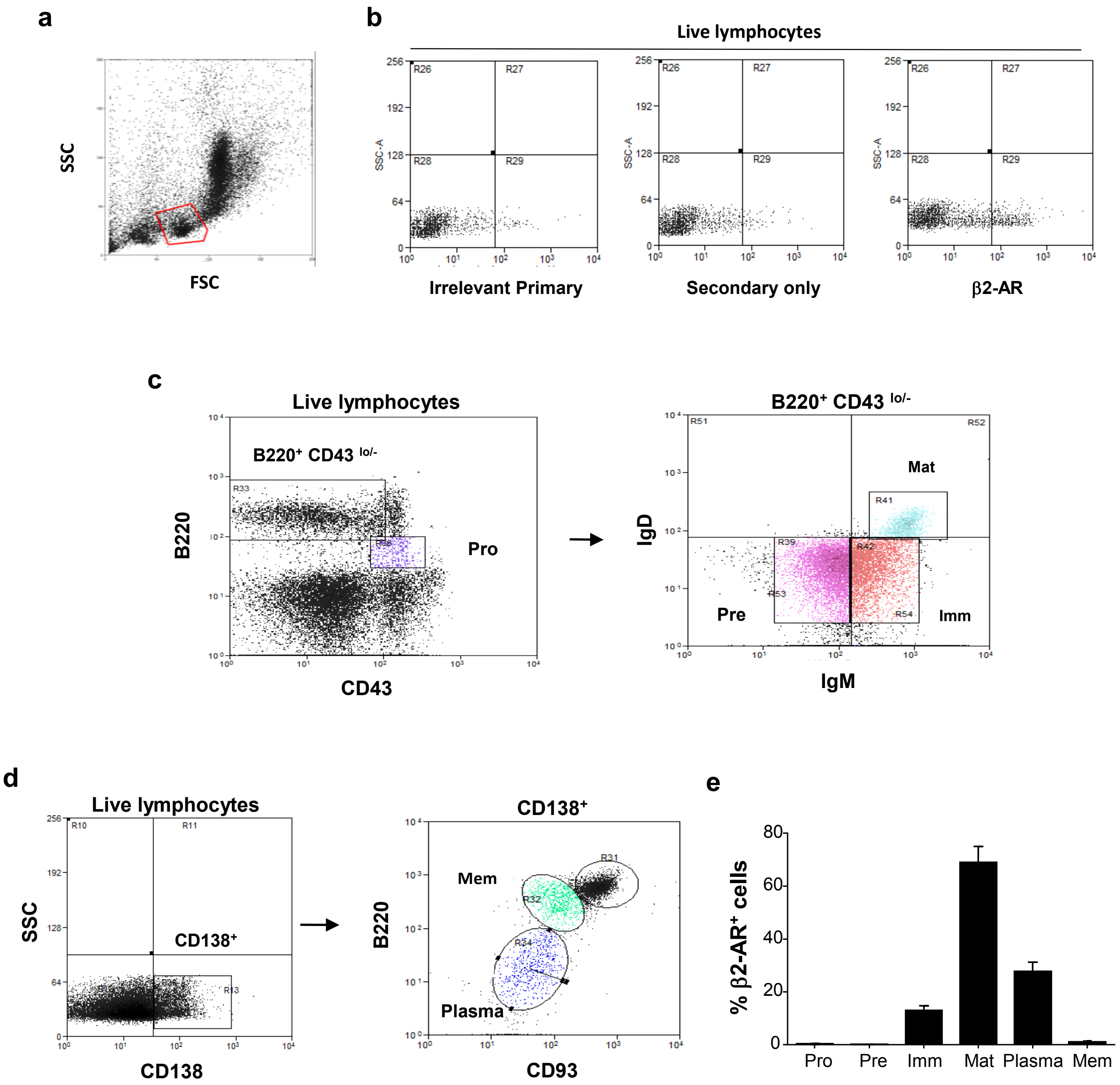


**Supplementary Figure 6**

A more stringent subdivision of bone marrow B cell subsets was used to confirm β2-adrenergic receptor (β2-AR) expression on B cells at different developmental stages. (**a**) Live lymphocytes were gated. (**b**) The threshold for positive β2-adrenergic receptor staining was set based on irrelevant primary and secondary only controls. (**c**) Live lymphocytes were plotted on CD43 and B220, Pro B cells were identified as $CD43^{+}B220^{lo}$. $CD43^{lo/-}B220^{+}$ cells were gated and plotted against IgM and IgD immunolabelling. Mature recirculating B cells (Mat) were identified as $IgM^{+}IgD^{+}$, immature B cells (Imm) were identified as $IgM^{+}IgD^{-}$ and pre-B cells were identified as $IgM^{-}IgD^{-}$. (**d**) $CD138^{+}$ cells were gated and plotted against CD93 and B220 immunolabelling. $CD93^{+}$ cells were excluded as immature B cells. $CD138^{+}CD93^{-}B220^{+}$ cells were identified as memory B cells (Mem) and $CD138^{+}$CD93-B220- cells were identified as plasma cells. (**e**) Expression of β2-AR on distinct bone marrow B cell subsets as identified in (c) and (d). The expression pattern was equivalent to that observed using an alternative gating strategy (see **Fig 6e**). n=4. Data show mean ± SD.

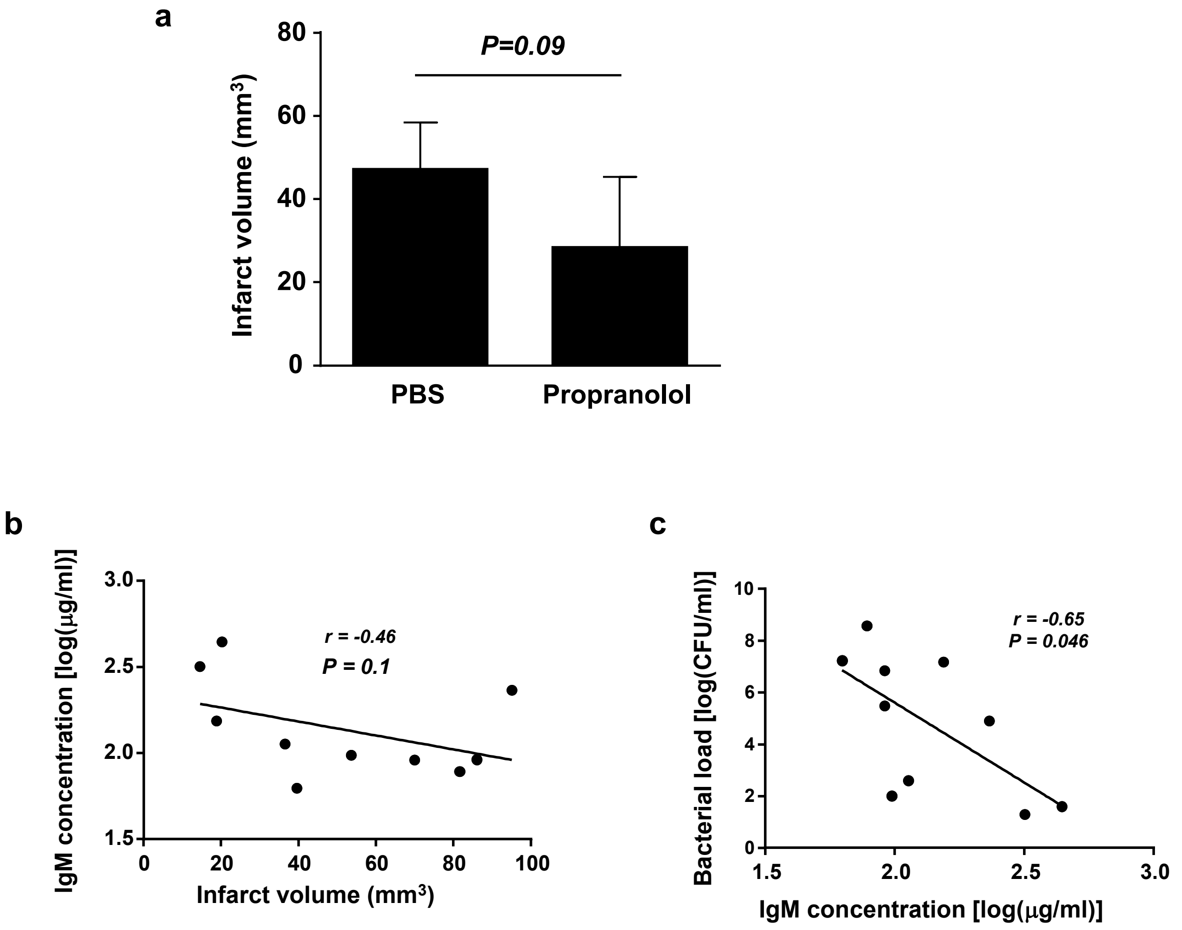


**Supplementary Figure 7**

(**a**) Infarct volume in the brains of animals treated with PBS or propranolol prior to and 4 h after MCAO. (*sham n=4, MCAO n=6*). (**b**) Relationship between plasma IgM concentration (log transformed) and infarct volume. (**c**) Relationship between plasma IgM concentration (log transformed) and lung bacterial load. Data show mean + SD; (**a**) unpaired *t*-test.

| | | Patients | |
|---|---|---|---|
| | Controls (n=36) | Infection (n=12) | No infection (n=22) |
| Median age (min, max), years | 70 (37, 91) | 70 (53, 91) | 68.5 (37, 86) |
| Male | 24 (66.7) | 9 (75.0) | 14 (63.6) |
| Significant atherosclerosis[a] | 17 (47.2) | 8 (66.7) | 8 (36.4) |
| Current or former smoker | 23 (63.9) | 8 (66.7) | 17 (77.3) |
| Hypertension | 11 (30.6) | 9 (75.0) | 14 (63.6) |
| Atrial fibrillation | 2 (5.6) | 2 (16.7) | 4 (18.2) |
| Diabetes mellitus | 6 (16.7) | 0 | 3 (13.6) |
| Ischaemic heart disease | 7 (19.4) | 2 (16.7) | 5 (22.7) |
| Previous stroke or transient ischaemic attack | 0 | 1 (8.3) | 6 (27.3) |
| Medications[b] | | | |
| Statin | 11 (30.6) | 2 (16.7) | 9 (40.9) |
| B-blocker | 7 (19.4) | 7 (58.3) | 8 (36.3) |
| Angiotensin Converting Enzyme Inhibitor | 3 (8.3) | 1 (8.3) | 2 (9.1) |
| Stroke characteristics | | | |
| Median NIHSS (min, max) | | 17.5 (9, 23) | 12.5 (3, 12.5) |
| Left hemisphere | | 6 (50.0) | 15 (68.2) |
| TACI | | 8 (66.7) | 6 (27.3) |
| PACI | | 4 (33.3) | 9 (40.9) |
| LACI | | 0 | 5 (22.7) |
| POCI | | 0 | 2 (9.1) |
| mRS at presentation | | 0.5 (0, 3) | 0 (0, 4) |

**Supplementary Table 1**
Baseline characteristics of patients with or without infections**.** All data as n (%), unless specified.
[a] defined as ≥50% stenosis of at least one internal carotid artery, and/or lowest ankle brachial pressure index of <0.92.
[b] receiving prior to admission or started during the first week.
**NIHSS**, National Institutes of Health Stroke Scale; **TACI,** Total Anterior Circulation Infarction; **PACI**, Partial Anterior Circulation Infarction;
**POCI**, Posterior Circulation Infarction; **LACI**, Lacunar infarction; **mRS**, modified Rankin Scale
